# Parathyroid-independent hypercalcemia as initial presentation of renal sarcoidosis with granulomatous interstitial nephritis: a case report

**DOI:** 10.3389/fendo.2025.1748226

**Published:** 2026-01-20

**Authors:** Liu Li, Yan Liu, Jishi Liu, Bin Yi, Xixi Qiao

**Affiliations:** Department of Nephrology, Central South University, Third Xiangya Hospital, Changsha, Hunan, China

**Keywords:** granulomatousinterstitial nephritis, hypercalcemia, renal biopsy, renal dysfunction, sarcoidosis

## Abstract

**Background:**

Sarcoidosis is a multisystem granulomatous disorder predominantly affecting the lungs. Renal sarcoidosis often presents diagnostic challenges, PTH-independent hypercalcemia may be a clinical feature.

**Case Report:**

A 48-year-old Asian female presented with persistent hypercalcemia (2.81 mmol/L), suppressed Parathyroid hormone PTH(1.24 pg/mL), and renal dysfunction (serum creatinine 2.02 mg/dL). Initial imaging showed mediastinal lymphadenopathy, 1.biopsy could not be performed as the size and location of the lymph nodes precluded safe conduct of the procedure, but bone marrow puncture and PET/CT excluded malignancy. A renal biopsy performed three months later demonstrated focal multinucleated giant cells and calcium deposits—confirming renal sarcoidosis. She was initiated on methylprednisolone (16 mg/day), leading to normalized calcium levels and improved renal function (serum creatinine stabilized at 1.1 mg/dL). However, despite strictly following the treatment plan, she still underwent ureteroscopy lithotripsy due to recurrent episodes of nephrolithiasis. Long-term follow-up after drug discontinuation showed that the patient’s calcium and PTH levels remained normal, while there was a mild impairment in renal function.

**Discussion:**

This case underscores the diagnostic difficulty of renal sarcoidosis, particularly in hypercalcemic patients without renal biopsy. Early use of corticosteroids can improve renal function, but complications like nephrolithiasis may still occur, so continuous monitoring is still necessary. A high index of suspicion and timely biopsy are essential for diagnosis and optimal management.

## Introduction

Sarcoidosis is a multisystem granulomatous disorder of unknown cause characterized by the formation of non-caseating epithelioid granulomas. The pathogenesis involves a complex interaction with environmental factors, genetic predispositions and immunological responses (1). This disease exhibits a worldwide distribution with considerable geographic variation in prevalence, ranging from 10 to 160 cases per 100,000 population. Notably Asian populations presents lower incidence rates (2, 3). The onset of sarcoidosis usually occurs between the ages of 20 and 50 years, with a peak between 20–39 years old, and women are 30% more likely to be affected than men. Clinical presentation is remarkably heterogeneous, including fatigue, weight loss, fever, cough, dyspnea, lymphadenopathy, pulmonary nodules, joint pain, skin lesions, uveitis, and liver, spleen, and kidneys (4, 5). The diagnosis requires careful exclusion of other granulomatous disorders while with characteristic histological findings.

Pulmonary involvement represents the prominent manifestation of sarcoidosis, occurring in approximately 90% of patients. Extrapulmonary disease is common, with renal involvement affecting 25-30% of cases. Other frequently affected organ systems include skin, eyes, liver, and peripheral lymph nodes (6, 7). Renal manifestations primarily present as two distinct pathological conditions: granulomatous interstitial nephritis (GIN) and hypercalcemia-related disorders. GIN constitutes the most common renal pathology, whereas hypercalcemia-induced complications occur in fewer than 10% of cases. The clinical presentation of renal sarcoidosis is often nonspecific, posing significant diagnostic difficulties, and also the clinical outcome is variable ranging from spontaneous remission to end-stage kidney disease (ESKD) and dialysis. Early diagnosis and prompt treatment with corticosteroids can improve the prognosis (1, 5).

In this report, we present a challenging case of renal sarcoidosis initially presenting with hypercalcemia and unexplained renal failure. The diagnosis was significantly delayed due to the lack of specific symptoms, with confirmation ultimately achieved three months after symptom onset.

## Clinical presentation

A 48-year-old Asian female was admitted on recurrent hypercalcemia and progressive renal impairment. The patient had no significant medical history or relevant family history. On initial presentation, she was admitted for hypercalcemia (serum calcium 2.81 mmol/L, normal range 2.2-2.7 mmol/L), anemia (hemoglobin 86 g/L, normal range 130–175 g/L), and impaired renal function (serum creatinine 2.02 mg/dL, baseline 0.46-0.91 mg/dL), accompanied by nonspecific abdominal discomfort. Physical examination was unremarkable. Diagnostic workup demonstrated suppressed intact parathyroid hormone (PTH 1.24 pg/mL, normal range 15–65 pg/mL) and low 25-hydroxyvitamin D level (17.71 ng/mL, normal range 20–100 ng/mL). Comprehensive evaluation including autoantibody testing, tumor markers, serum and urine protein electrophoresis with immunofixation revealed no abnormalities. Neck ultrasound identified a 5*2 mm hypoechoic nodule posterior to the inferior right parathyroid gland, suggestive of parathyroid hyperplasia. Thoracic CT scan showed enlarged lymph nodes in the left pulmonary hilum and mediastinum, while whole-body bone scintigraphy was negative. Bone marrow biopsy demonstrated normocellular hematopoiesis without evidence of malignancy or dysplastic changes. The patient’s hypercalcemia responded temporarily to intravenous hydration and calcitonin therapy, following which she was discharged with outpatient follow-up arrangements for further diagnostic evaluation.

**Figure 1:**
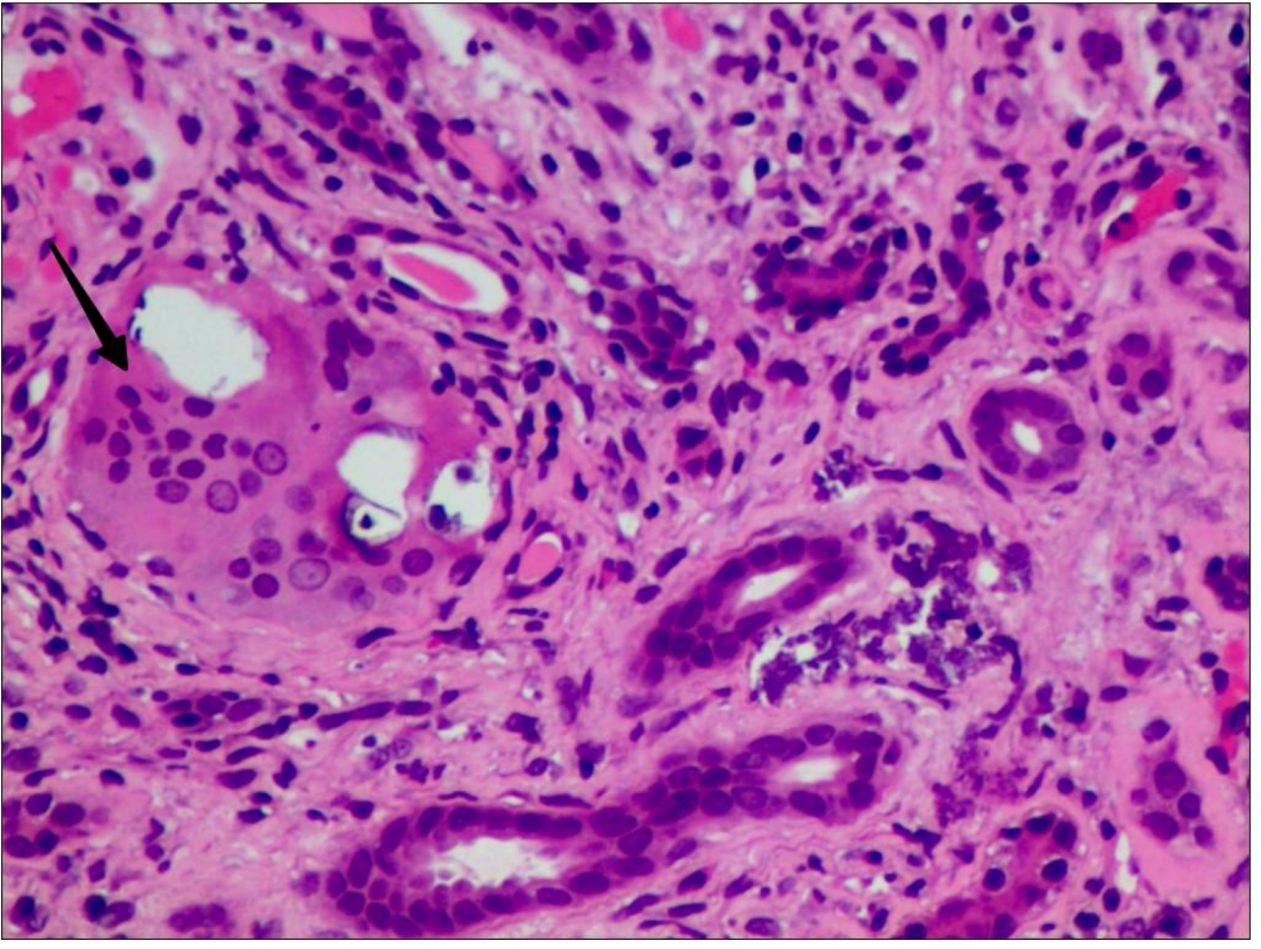
Renal biopsy: Interstitial granuloma with multinucleated giant cells (arrow) (H&E, X600) H&E: hematoxylin and eosin.

Three months later, the patient was readmitted due to worsening hypercalcemia and progressive renal impairment. While hemodynamically stable with unremarkable physical examination, laboratory tests revealed elevated serum calcium (2.87 mmol/L), worsening renal function (serum creatinine 2.40 mg/dL), persistently suppressed PTH (2.8 pg/mL), and low 25-hydroxyvitamin D (19.42 pg/mL). Proteinuria was noted with 24-hour urine protein excretion of 786 mg. FDG-PET/CT demonstrated multiple enlarged lymph nodes with variable sizes in bilateral cervical, supraclavicular, hilar, mediastinal, axillary, paraaortic, iliac, and inguinal regions, all suggestive of reactive hyperplasia rather than malignancy. Following multidisciplinary consultation with surgical and interventional radiology teams, lymph node biopsy was deemed technically challenging due to the deep location and small size of the lymph nodes. Given the persistent hypercalcemia and progressive renal dysfunction, a renal biopsy was performed, revealing mild mesangial proliferative glomerulopathy accompanied by multifocal tubular atrophy, thickened basement membranes and interstitial fibrosis, with focal tubular epithelial brush border loss and granular degeneration. The interstitium shows occasional basophilic crystalline deposits and focal multinucleated giant cells (**Figure 1**). These characteristic findings supported the diagnosis of renal sarcoidosis. The patient was initiated on moderate-dose corticosteroid therapy and subsequently discharged with close follow-up arrangements.

**Figure 2:**
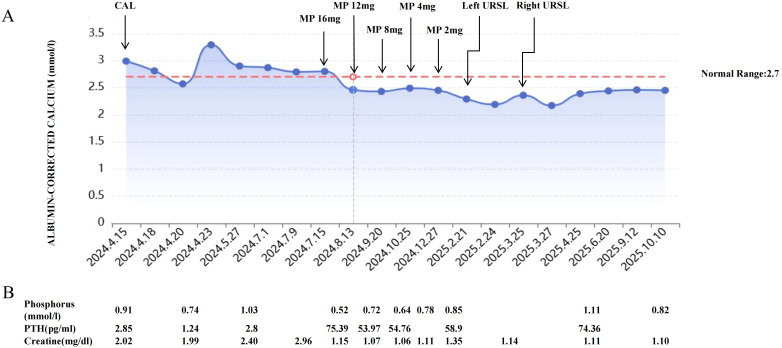
**(A)** Calcium levels under therapy. CAL, calcitonin; MP, Methylprednisolone; URSL,Ureteroscopic Lithotripsy. **(B)** Selected PTH, phosphate and creatinine levels. PTH, parathyroid hormone.

The patient was discharged with scheduled outpatient follow-up. **Figure 2** illustrates the dynamic changes in serum calcium and creatinine levels before and after corticosteroid therapy. One month post-treatment, both calcium and PTH levels normalized, while serum creatinine significantly decreased. After 6 months of treatment, the steroids were successfully tapered and discontinued while maintaining disease stability. Seven months later, the patient was readmitted to the urology department due to acute flank pain caused by nephrolithiasis, ultimately undergoing two unilateral ureteroscopic lithotripsy procedures. Following steroid withdrawal, the patient’s condition remained stable, though mild renal dysfunction persisted.

## Discussion

Sarcoidosis represents an uncommon systemic disorder, with renal involvement being particularly rare. The manifestation of hypercalcemia as the primary clinical presentation is exceptionally uncommon and has been associated with poorer prognostic outcomes (8, 9). In the current case, the patient initially presented with hypercalcemia and renal dysfunction. Following comprehensive exclusion of neoplastic etiologies, the definitive diagnosis of renal sarcoidosis was established through renal biopsy findings, which demonstrated characteristic multinucleated giant cells. Notably, the patient exhibited marked clinical improvement following initiation of corticosteroid therapy, with normalization of calcium homeostasis and stabilization of renal function.

Berline et al. in an analysis of 52 GIN patients reported that urinalysis findings included sterile pyuria (32.7%), hematuria (21.2%), glycosuria (11.5%), and hypercalciuria (7.7%). Notably, most patients exhibiting proteinuria levels <1 g/day, while massive proteinuria is relatively uncommon (approximately 2.5% of cases), which may be attributed to renal tubular dysfunction (10). A pathological analysis of 9,779 renal biopsy specimens revealed only 46 cases (0.5%) of GIN (11). Although GIN is considered the most pathognomonic renal manifestation of sarcoidosis, histological examination fails to detect granulomas in 25-44% of cases, presenting a significant diagnostic challenge (12). Histologically, the disease manifests as organized aggregates of CD4+ T lymphocytes, B-cell clusters, and activated macrophages exhibiting M1 polarization that may coalesce to form multinucleated giant cells. These granulomas may or may not be surrounded by a distinct lymphocytic cuff. The presence of giant cells, extensive interstitial infiltrates, and interstitial fibrosis reveals a poor prognosis of GIN (13, 14). Hypercalcemia develops in approximately 10-20% of patients with sarcoidosis, primarily mediated by extrarenal dysregulation of vitamin D metabolism. Activated granuloma macrophages enhance the conversion of vitamin D to its active form (1,25-dihydroxyvitamin D), leading to increased intestinal calcium absorption and suppression of PTH secretion. This mechanism is associated with a worse prognosis (5). Hypercalcemia may be asymptomatic or manifest with potentially life-threatening organ dysfunction like neuromuscular, cardiovascular, or gastrointestinal complications, tissue calcifications and renal damage. Hypercalcemia-induced acute kidney injury (AKI) primarily results from afferent arteriolar vasoconstriction and impaired renal autoregulation, while chronic hypercalcemia may lead to irreversible complications including nephrocalcinosis, nephrolithiasis, and chronic tubulointerstitial nephritis. The severity of renal dysfunction typically correlates with both the magnitude and duration of calcium elevation (9, 15).

The diagnosis of sarcoidosis is based on three major criteria: consistent and adequate clinical presentation, demonstration of the presence of non-caseating granulomas in one or more tissue samples and the exclusion of other causes. It should be emphasized that non-caseating granulomas, while characteristic, are not pathognomonic for sarcoidosis. The main differential diagnoses include infectious etiologies, drug-induced granulomatous reactions, common variable immunodeficiency [particularly common variable immunodeficiency (CVID)], inflammatory diseases [granulomatosis with polyangiitis (GPA) and granulomatosis with eosinophilia and polyangiitis (EGPA)], and neoplastic disorders (4, 6). Keijsers et al. found that FDG-PET was positive in 34 of 36 patients with sarcoidosis assessed, reported a high sensitivity (94%) (16). Beyond lesion mapping, FDG-PET provides quantitative assessment of inflammatory activity and serves as an objective biomarker for treatment response monitoring (17). The assessment of renal involvement by FDG PET/CT presents unique diagnostic challenges owing to the physiological urinary excretion of 18F-FDG. A case series reported that kidney lesions could not be detected by FDG PET/CT. The physiological tracer uptake in the kidney interferes with the identification of tissue lesions. Therefore, the sensitivity of PET/CT may not be high enough for patients having renal lesions (18). Elevated serum angiotensin-converting enzyme (ACE) levels are well-documented in sarcoidosis, with reported detection rates ranging from 40% to 90%. The enzyme is produced by granuloma cells (epithelioid cells, giant cells, macrophages). However, its diagnostic value is limited by 40% false-negative and 10% false-positive rates. Note that other granulomatous diseases may also increase ACE, requiring differential diagnosis (19).

Systematic reviews among patients with sarcoidosis identified 6% hypercalcemia, 84% had a low 25-(OH) vitamin D level and 11% had a high 1,25-(OH)2 vitamin D level (19). Normal hydroxylation of 25-hydroxyvitamin D to 1,25-dihydroxy vitamin D occurs in renal proximal tubular cells by 1-αhydroxylase. In patients with sarcoidosis, activated macrophages expressing 1-αhydroxylase, increases conversion of 25-hydroxyvitamin D into biologically active vitamin D, which enhances both intestinal absorption of dietary calcium and osteoclast activity and bony reabsorption, thus predisposing to hypercalcemia. The consequent vitamin D dysregulation and hypercalcemia suppress parathyroid hormone secretion (6).

Corticosteroids was the first-line therapy for renal sarcoidosis, though optimal dosing and treatment duration lack standardization. Most patients demonstrate significant renal response (defined as ≥50% eGFR improvement) within the first month of treatment, while failure to achieve this early response often predicts poorer outcomes (20). For steroid-refractory or intolerant cases, alternative immunosuppressants including azathioprine, mycophenolate mofetil, or TNF-αinhibitors may be considered (1, 5).

## Conclusion

Renal involvement in sarcoidosis frequently remains asymptomatic for prolonged periods. In patients presenting with unexplained hypercalcemia with renal dysfunction, especially in PTH-independent conditions, renal sarcoidosis should be considered.
